# The impacts of various green space types on the adiposity of undergraduate students: a nationwide quasi-experimental study

**DOI:** 10.1186/s12942-025-00402-0

**Published:** 2025-07-17

**Authors:** Jing Wen, Yi Lu, Xiangfen Cui, Weina Kong, Kai Shentu, Haoran Yang

**Affiliations:** 1 The Center for Modern Chinese City Studies, East China Normal University, Shanghai, 200241 China; 2 Zhejiang Economic Information Center, Hangzhou, 310006 Zhejiang China; 3 Department of Architecture and Civil Engineering, City University of Hong Kong, Hong Kong, China; 4 Faculty of Environmental Science and Engineering, Kunming University of Science and Technology, Kunming, 650500 China; 5 Antai College of Economics & Management, Shanghai Jiao Tong University, Shanghai, 200030 China; 6 School of Geographic Sciences, East China Normal University, Shanghai, 200241 China

**Keywords:** Green space, BMI, physical activity, Difference-in-differences model, Quasi-experiment

## Abstract

Green spaces provide diverse health benefits, and provision of green spaces is often linked to lower incidences of adiposity. Undergraduates, who are at a transitional stage of development, represent a critical population for obesity prevention. However, recent studies suggest that the health effects of green space may vary by type. Furthermore, inferring any causal relationship between green spaces and adiposity using a cross-sectional research design remains challenging. To address these issues, this study utilized a large, representative sample of 21,990 undergraduates from 89 universities across 29 provinces in China, and employed a quasi-experimental approach to explore the impacts of specific green space types on body mass index (BMI). Propensity score matching was used to make the students who were influenced by green spaces comparable to those who were not. A difference-in-differences model was applied to estimate the causal effects of three types of green spaces (trees, bushes, and grass) on BMI. To further explore the underlying mechanisms, we examined two potential mediators: energy expenditure (physical activity) and energy intake (unhealthy food consumption). The results revealed that trees had a negative impact on BMI, whereas bushes and grass had no significant effect. Physical activity serves as a significant mediator linking tree exposure to adiposity changes, while unhealthy food intake showed no statistically significant mediation effect. In the stratified analysis, trees had significantly negative effects only on males. These findings highlight the importance of distinguishing green space types and provide causal evidence linking tree exposure to reduced BMI among undergraduates.

**Supplementary Information:**

The online version contains supplementary material available at 10.1186/s12942-025-00402-0.

## Introduction

Adiposity, the accumulation of excess body fat, is associated with various health risks. When body fat levels become particularly high, this can lead to overweight or obesity. Globally, the prevalence of adiposity in adults has tripled to 13% between 1975 and 2016, and the prevalence of overweight and obesity among individuals aged 5–19 has risen considerably from 4% in 1975 to over 18% in 2016 (WHO, 2021). In China, the proportion of obese children and adolescents jumped from 1.2% in 1985 to 23.4% in 2019 [15]. Thus, effective and immediate actions are required to reverse or slow the trend of obesity, as it has become a global health concern [44].

University students represent a unique population with distinct risk factors for adiposity compared to other age groups. Studies have shown that students often gain weight after entering university [64]. One possible explanation is their higher metabolic rate during this developmental stage, which may increase food intake demands [3]. In addition, undergraduates are more likely to adopt unhealthy lifestyle behaviors such as frequent consumption of fast food and snacks [54] and prolonged sedentary time [41]. Given the increasing prevalence of obesity and the specific behavioral risks among this group, developing targeted interventions for university students is essential for promoting their long-term health and well-being.

Integrating health into the process of urban planning and urban design helps achieve the coordinated development of cities and health [6]. According to social-ecological models, the urban built environment can stimulate healthy behaviors and outcomes [20]. Green spaces are a crucial environmental factor potentially affecting health [46, 66]. Exposure to green spaces could promote physical activity, improve air quality, and facilitate social interaction, so that reduce adiposity [16, 20]. For example, Trees can promote walking by providing shade and improving thermal comfort [1, 63]. Bushes may be invisible, inaccessible, and unappealing to users, such as green strips along highways or overgrown areas near abandoned buildings [76]. Grass areas often support social interaction and relaxation, which are linked to psychological well-being [4, 29]. However, some studies have reported conflicting or null findings regarding the relationship between green space exposure and adiposity. For instance, Wilhelmsen et al. [68] found that the percentage of overweight and obese adolescents increased significantly with a higher proportion of surrounding green areas. In contrast, Mowafi et al. [42], using multilevel modeling in Cairo, reported no significant association between green space exposure and BMI among adults. These inconsistencies may stem from several factors, including the cross-sectional designs that limit causal inference, the reliance on coarse greenness measures that overlook green space diversity, and the heterogeneity across population subgroups in terms of age, gender, and behavioral patterns.

First, most studies used a cross-sectional research design that makes it arduous to detect any causal relationships due to inherent limitations such as residential self-selection bias. Self-selection bias is mainly caused by non-randomly selected samples [25]. Due to individual’s preference for residential locations, people are not randomly assigned to where they live. Hence, the observed association between green spaces and adiposity can be confounded by the self-selection effect [17, 23] and consequently explained by other individual factors rather than being a true causal effect. To mitigate self-selection bias, it has been proposed to focus on specific groups of people with little freedom in selecting their locations, such as those living in public housing estates or university campuses [55, 73, 75]. Chinese undergraduates spend the majority of their time on campus and have little freedom in residential choices [75]. In this context, green spaces in the campus environment can be considered to have a true causal effect on the health outcomes of college students.

On the methodological front, some studies have utilized a quasi-experimental research framework to determine the causality between green spaces and public health [70, 71]. Quasi-experiments are typically conducted in response to a specific intervention. Thus, quasi-experiments require the identification of an intervention, a treatment group consisting of participants impacted by the intervention, and a comparable control group. By comparing health changes of the treatment and control groups, endogeneity effects caused by unmeasured confounders are mitigated [70]. However, there could still be some non-random bias between the treatment and control groups, making it difficult to determine the true effect of the treatment. Propensity score matching (PSM) is an effective method for making the treatment and control groups comparable by balancing the distribution of observed confounding variables (i.e., gender, age, exposure to the built environment) between the two groups [50, 58]. We will further discuss this method in Sect. "Covariates".

Second, previous studies often used the overall greenness level, such as the normalized difference vegetation index (NDVI), while overlooking diverse types of green spaces. However, different green space types possess distinct ecological and structural characteristics, and may contribute to health outcomes [21, 38, 47]. Wu et al. [69] pointed out that urban green space influences public health not only through vegetation cover but also via vegetation composition and dominant species. In recent years, an increasing number of studies have begun to explore the heterogeneous health effects of specific green space types, such as trees, grass, forests, rangelands, agricultural lands, and wetlands [5, 30]. For instance, studies have found that tree canopy has a stronger protective effect on sleep quality than grass [5], forest cover is consistently associated with lower perceived stress [35], and shrubs and grass appear to have a greater impact on mental health compared to general public green space access [30]. One possible reason for these differences is that various green space types influence behavior differently. For example, moderate to vigorous physical activity tends to be higher in areas with greater tree canopy but not with open grassland [18]. Therefore, it is essential to move beyond aggregate greenness measures and delve into the distinct health impacts associated with different types of green spaces.

To better understand how these diverse green space types influence health outcomes, it is also important to examine the mechanisms underlying the association between diverse types of green spaces and adiposity. Energy expenditure and intake serve as potential mediators of the built environment-adiposity association [27, 40, 74]. A growing body of literature suggests that green spaces can promote energy expenditure by stimulating physical activity (PA) [16, 71]. This pathway is particularly relevant in university settings, where undergraduates often engage in short bouts of PA between classes. Tree-covered areas that offer shade and walkability may be especially conducive to such activity [1, 63]. In contrast, bushes and grass areas are often less accessible or not designed for walking, as students typically cannot walk through densely planted shrubs or cross lawns in urban campus contexts [77]. However, few studies have explicitly tested this mediating pathway among college students, or examined how different types of green space affect PA levels.

As for energy intake, green spaces may help reduce the intensity and frequency of unhealthy food cravings [39], especially the choice of “reward drink” [13, 31]. Meanwhile, it provides opportunities for outdoor dining activities, such as picnics [43]. These pathways highlight how green spaces can create a more health-conscious eating environment by subtly encouraging healthier food behaviors. Therefore, it is essential to examine how different types of green spaces contribute to mechanisms like energy intake and expenditure.

Third, the association between green spaces and adiposity may vary among different population subgroups, particularly in terms of age and gender differences, which can influence the usage of green spaces [38, 53]. For example, women often prioritize safety concerns [11] and tend to reduce their PA time in areas with more green spaces [19]. Most studies have focused on adults [2, 61] while some have examined children and adolescents [67, 68]. However, there is limited research on young adults, particularly college students, who warrant special attention due to the impact of body shape and weight status on their romantic relationships [9] and employment opportunities and wages [22]. Thus, investigating the potential benefits of green spaces for college students is critical.

To address these issues, our study focuses on undergraduate students in China who reside in campus dormitories and have little freedom to select where they live to mitigate residential self-selection bias [72, 73, 75]. Then, we infer the causal links, the underlying mechanisms, and heterogeneous effects between three types of diverse green spaces (i.e., trees, bushes, and grasses) and the body mass index (BMI) of undergraduate students. Specifically, we considered first-year students as the pre-intervention group, unexposed to campus green spaces, and sophomores and above as the post-intervention group, exposed for at least one year. By comparing BMI changes between these groups, we determined the net impact of green space exposure [24, 34, 70]. To address non-random bias, we employed propensity score matching (PSM) to ensure group comparability [50, 58], followed by a difference-in-differences (DID) regression based on the propensity scores to estimate the causal impact of different green spaces on BMI.

## Material and methods

### Study setting and sample

**Fig. 1:**
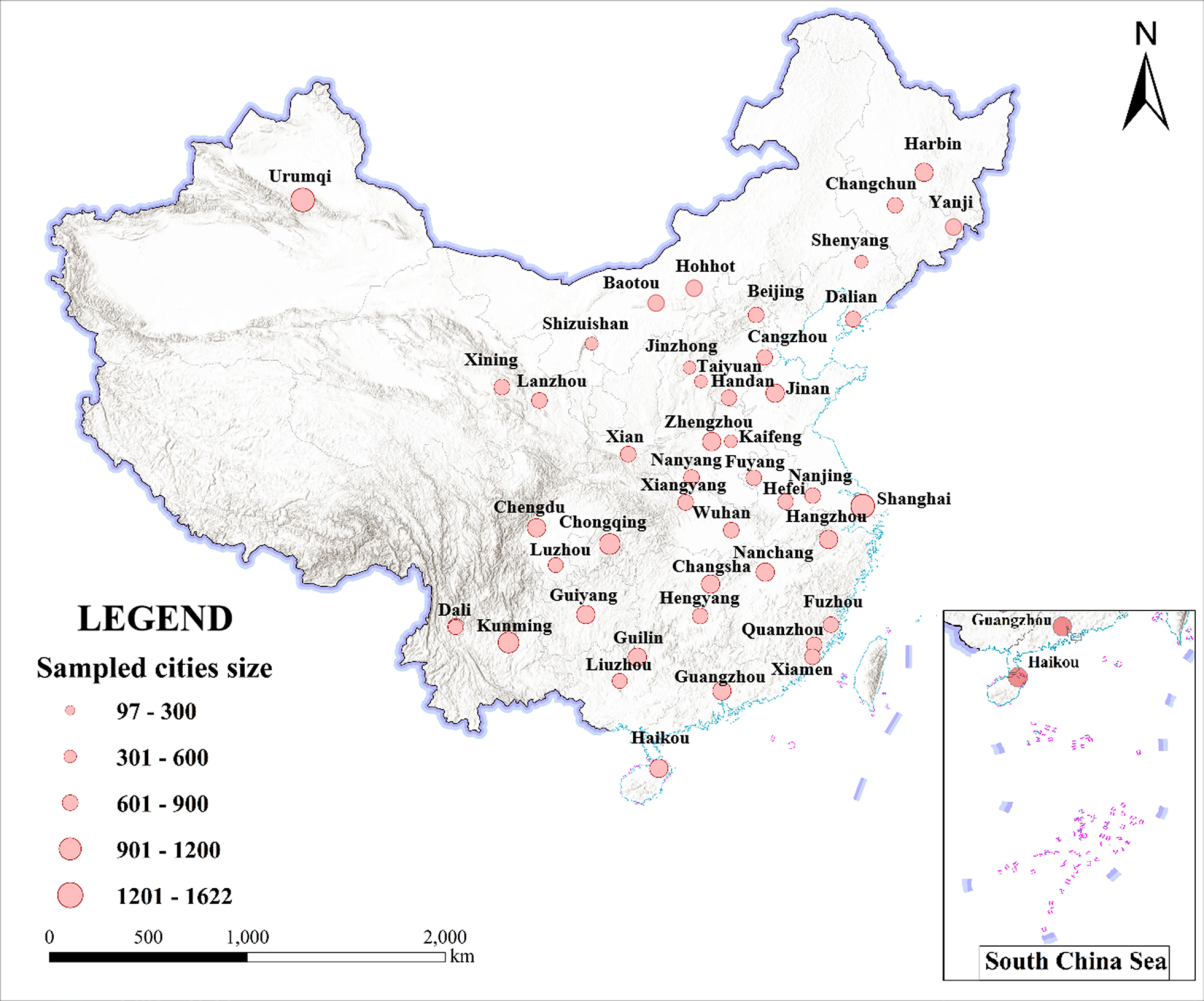
Spatial distribution and sample size of undergraduate students participating in this study

This study utilized nationwide survey data obtained from the First Affiliated Hospital of Kunming Medical University (Ethical number: 2018-L-25). The survey was conducted from September 19 to 26, 2018, by medical professionals from hospitals. The eligibility criteria for participants involved a multiple-stage stratified sampling procedure: (a) The majority of provinces/municipalities (29 in total, excluding Tianjin and Tibet) in mainland China participated in the survey. (b) Two to four campuses in each province/municipality were randomly selected. (c) A total of 300 to 700 undergraduate students were selected from each campus based on the probabilities proportional to the population size of undergraduate students. The initial sample included 23,437 undergraduate students from mainland China (Fig. 1). After excluding missing values of Hukou status (n = 1428) and 19 samples that did not meet the requirements for propensity score matching [57], the valid sample consisted of 21,990 respondents from 89 campuses in 29 provinces.

Data collection involved the use of a questionnaire and physical examination. Students provided active informed consent. Demographic and socioeconomic attributes were collected through a self-reported questionnaire, while health-related behaviors were obtained via face-to-face interviews. Adiposity status was measured by healthcare professionals.

Environmental exposure data were gathered within a 1,000 m buffer surrounding the centroid of each campus using a GIS-derived approach [68]. The 1,000 m buffer was selected as it encompassed the daily activities of the majority of students. This choice is supported by the fact that the average length of the longest radius from a campus centroid to its boundary is 527 m. Additionally, a 1,000 m distance is commonly regarded as suitable for walking [37]. For college students, when the distance is less than 1,000 m, most students choose to walk or bike [75]. In the context of our study, we focused on walking behavior, as it is more commonly observed and more consistently associated with energy expenditure in short-distance settings on university campuses.

## Health indicator data

The BMI, which is calculated as weight divided by height squared, is commonly used as a proxy for adiposity. In this study, the height and body weight of participants were measured by trained medical staff using calibrated equipment. The specific models and brands of the equipment may vary across different hospitals, but all equipment used in the study was authorized and calibrated by the respective hospitals.

## Green space data

To assess campus green space exposure, we adopted a GIS-derived land cover approach, which allows for consistent spatial coverage and scalable application across multiple campuses. This method ensures that green spaces within and around each campus are fully captured. In particular, we used the local climate zone (LCZ) classification system, which describes land cover, structure, and urban morphology [59]. LCZ maps provide more detailed information about different types of green spaces compared to aggregated green space data. Moreover, LCZ data is processed by freely available Landsat 8 images and SAGA GIS accessible to anyone to refer to, share, and process by the standard World Urban Database and Access Portal Tools (WUDAPT) [7, 65]; thus, LCZ data has been widely utilized in urban studies [56, 60]. The LCZ classification consists of 17 classes, including ten built types and seven natural types, offering a practical and cost-effective approach applicable to any region [59]. In this study, we focused on three specific LCZ classes: trees (LCZ A and B), bushes (LCZ C), and grass (LCZ D) to examine their impact on adiposity. We used LCZ data from 2018, consistent with the timing of the survey. This is considered appropriate because LCZ classifications reflect stable land cover [59], and campus environments in China have shown limited change in recent years [36].

Following the WUDAPT workflow [48], we obtained LCZ maps for the 89 campuses within a buffer radius of 1,000 m provided by Hong Kong University [48, 56]. In detail, the 30 m resolution Landsat 8 level 0 images were obtained from the U.S. Geological Survey (http://glovis.usgs.gov), and then training polygons of each LCZ type were created following the WUDAPT method and classifying each image pixel into one LCZ type in SAGA GIS [48]. A representative case (Figure S1, supplementary material) illustrates how different green space types were spatially identified and classified for analysis.

## Mediators

The balance between energy intake and energy expenditure plays a role in modifying adiposity [27]. Therefore, both energy expenditure and energy intake are considered potential pathways through which the built environment can influence adiposity. Energy expenditure in green space considerably reduces the health risk of non-communicable diseases [2]. In this study, the duration of outdoor walking was used as a proxy for energy expenditure. The walking duration was measured as follows (Eq. 1):

1 $$Expenditure=Frequecy*Duration$$

where $Expenditure$ represented the duration of walking per week. According to Yin et al. [74], $Frequecy$ represented the frequency of walking with six response categories, ranging from none (0) to every day (1), including once per month (1/30), once per two weeks (1/14), once per week (1/7), and two or three times per week (2.5/7). $Duration$ represented the duration of each exercise session, categorized into ≤ 1 h (0.5), 1–2 h (1.5), 2–4 h (3), and > 4 h (5).

Furthermore, green spaces may be associated with energy intake. Previous research has shown that green spaces are inversely associated with the strength and frequency of unhealthy food cravings, suggesting their potential to reduce the intake of unhealthy foods [13, 39]. Additionally, green spaces provide opportunities for outdoor dietary activities, such as picnics [43], which can contribute to food intake. In this study, we measured energy intake through unhealthy food consumption, including salty, oily, and sweet foods, which are strongly associated with obesity [40]. Participants were asked about their dietary habits, and scores ranging from none (0) to very much (5) were assigned for each food category. The unhealthy food intake score was calculated by summing the scores for these food categories.

## Covariates

To avoid the confounding influence of the built environment (BE) features, SES and demographic characteristics on adiposity, several variables were controlled, including population density [73], street connectivity [14], bus-stop density [73], fast-food density [16], PA [73], living costs [73], smoking [68], alcohol [61], age [45], gender [45], ethnicity [68], and Hukou type [61]. The definitions and data sources of these variables are provided in Table 1.

**Table 1:** Definitions and data sources

	Variables	Definition	Data source
Adiposity	BMI	Weight divided by height squared	Medical examination measured by healthcare professionals
Green spaces	Trees	Dense and scattered trees	LCZ map in 2018
	Bush	Bushes, shrubs, and short, woody trees	
	Grass	Grass or herbaceous plants/crops	
	Exposure	A dummy variable. ${Exposure }_{i}$ is a dummy variable of green space exposure; ${Exposure }_{i}$ = 1 refers to sophomores and above (i.e., those influenced by green spaces). ${Exposure }_{i}$= 0 refers to first years (i.e., those not influenced by green spaces)	Survey
BE features	Population density	Population divided by the buffer area	Worldpop in 2018, with 100 * 100 m resolution
	Street connectivity	The number of intersections within the buffer	Open Street Map in 2018
	Bus-stop density	The number of bus stops is divided by the buffer area	Amap in 2019
	Fast-food density	The number of fast-food restaurants is divided by the buffer area	
Mediators	PA	Weekly time (minutes/week) spent on active travel	Survey
	Food	The frequency of unhealthy food intake	
SES and demographic characteristics	Living costs	Respondent’s monthly living expenditure level (< 1,000 / 1,000–2,000 / > 2,000 RMB)	Survey
	Alcohol	If the respondent is or has been a drinker, this variable = 1, 0 otherwise	
	Smoking	If the respondent is or has been a smoker, this variable = 1, 0 otherwise	
	Age	The respondent’s age	
	Gender	If the respondent is male, this variable = 1, 0 otherwise	
	Type of Hukou	If the respondent’s household registration is in urban areas, this variable = 1, 0 otherwise	
	Ethnicity	If the respondent is of Han ethnicity, this variable = 1, 0 otherwise	

## Data analysis

We first categorized the respondents into two groups according to their length of exposure: those exposed to the environment (sophomore and above, Exposure = 1) and those not exposed (first years, Exposure = 0). We assumed that the body weight of the first years was not influenced by campus green spaces as they had just enrolled when the data was collected and the exposure duration was too short to influence their BMI. Hence, first-years were regarded as the pre-intervention group. Conversely, the body weight of sophomores and above was influenced by green spaces; therefore, we regarded them as the post-intervention group. In summary, taking advantage of the characteristics of our dataset, we converted cross-sectional data into quasi-panel data and a quasi-experiment was established to detect the causality between diverse green space types and BMI.

The ideal approach to explore this is to compare the effect of exposure to green spaces with the effect of non-exposure based on the same samples. However, it is impossible to test the causal effect of green spaces with two identical sample sets. To make first-years and sophomores and above comparable, we employed PSM that matched the freshmen with sophomores and above based on two types of factors (i.e., BE, SES and demographic characteristics). We further adopted DID to estimate the net impact of diverse types of green spaces on BMI. In detail, we first calculated a propensity score using the kernel matching method of PSM as benchmark results and the radius matching method as a robustness check. Then we used the propensity scores as weights and conducted the weighted regression in the DID stage.

Specifically, considering the random grouping and heterogeneity of the individuals, we adopted the PSM method to match freshmen and sophomores and above using BE features and seven SES and demographic characteristics [58]. First, we calculated the propensity score based on logit regression:

2 $$p\left({respondent}_{b}^{l}\right)=pr\left({D}_{l}=1|{respondent}_{b}^{l}\right)=\frac{\text{exp}({\delta }_{b}{respondent}_{b}^{l})}{1+\text{exp}({\delta }_{b}{respondent}_{b}^{l})}$$

where ${respondent}_{b}^{l}$ represents the observable characteristics of the individual $l$ within group $b$, $p\left({respondent}_{b}^{l}\right)$ denotes the propensity score, and ${D}_{l}$ is a dummy variable that equals 1 if the individual is exposed to green space and 0 otherwise. ${\delta }_{b}$ is the corresponding coefficient..

Kernel matching, a non-parametric matching method, was used to match first-years and sophomores and above. By using a weighted average that was inversely proportional to the distance between the propensity scores of first years and sophomores and above, the treatment units were matched with all control units [8]. In general, most control units can be utilized without wasting data through kernel matching. We also tried Gaussian and Biweight kernel matching functions in Sect. "Robustness check". Kernel matching can be expressed by the following equation:

3 $$\omega \left(a,b\right)=\frac{K(\frac{{p}_{a}-{p}_{b}}{R})}{\sum K(\frac{{p}_{a}-{p}_{b}}{R})}$$

where $a$ represents a respondent in the treatment group (i.e., exposed to a greater level of green space), and $b$ represents a respondent in the control group (i.e., exposed to a lesser level of green space). The terms ${p}_{a}$ and ${p}_{b}$ represent the propensity scores of the respondents exposed to the greater green space area a and the lesser green space area b, $\omega \left(a,b\right)$ is the weight used in kernel matching, and R is a bandwidth parameter. The type of kernel function was Epanechnikov. The bandwidth parameter R was set to 0.06 times the standard deviation of the estimated propensity scores, following the default configuration in the Stata psmatch2 command. This setting was consistently applied across all kernel functions examined in Sect. "Robustness check".

To compare whether exposure to high levels of green spaces affected BMI, we further divided respondents into two categories: the treatment group in green space areas higher than the median values, and the control group in green space areas lower than the median values. To avoid potential multicollinearity among the interaction terms and to isolate the specific effect of each green space type, we estimated separate models for trees, bushes, and grass. Accordingly, we constructed three DID models to estimate causal effects using cross-sectional data. The models are specified as follows:

4 $${BMI}_{i}={\beta }_{0}+{\beta }_{1}{Htree}_{i}+{\beta }_{2}{Exposure }_{i}+{\beta }_{1}{Htree}_{i}*{Exposure }_{i}+\sum {\tau }_{j}*{X}_{ji}+{\varepsilon }_{i}$$

5 $${BMI}_{i}={\beta }_{0}+{\beta }_{1}{Hbush}_{i}+{\beta }_{2}{Exposure }_{i}+{\beta }_{1}{Hbush}_{i}*{Exposure }_{i}+\sum {\tau }_{j}*{X}_{ji}+{\varepsilon }_{i}$$

6 $${BMI}_{i}={\beta }_{0}+{\beta }_{1}{Hgrass}_{i}+{\beta }_{2}{Exposure }_{i}+{\beta }_{1}{Hgrass}_{i}*{Exposure }_{i}+\sum {\tau }_{j}*{X}_{ji}+{\varepsilon }_{i}$$

where ${BMI}_{i}$ represents the BMI score of respondent *i*. ${Htree}_{i}$ is a dummy variable of the level of trees in the buffer (high vs. low): ${Htree}_{i}$= 1 if the value is above the medium; otherwise, ${Htree}_{i}$= 0 in Eq. (4). Similarly, we used ${Hbush}_{i}$ and ${Hgrass}_{i}$ in Eq. (5) and Eq. (6), respectively. ${Exposure}_{i}$ is a dummy variable of green space exposure: ${Exposure}_{i}$= 1 refers to sophomores and above (i.e., those influenced by green spaces), ${Exposure}_{i}$= 0 refers to first years (i.e., those not influenced by green spaces). ${X}_{ji}$ refers to the matrix vector of the covariates, and ${\varepsilon }_{i}$ refers to the stochastic disturbance term.

All students in this study came from 89 campuses, possibly leading to within-groups correlated homogeneity errors [28]. Accounting for these errors caused by within-campus homogeneity, all the regressions were based on the cluster-robust standard errors. In this regard, we can safely assume that students within the same campus are independent of each other. All the results were analyzed using Stata 17.0.

## Results

### Descriptive statistics

Table 2 describes the characteristics of first-years and sophomores and above. The average age of the first years was 18.479 years (SD = ± 1.182), while the average age of sophomores and above was 20.578 years (SD = ± 1.572). The average BMI of sophomores and above was very close to that of the first years but had a larger range. The t-test results indicated a significant variance between first-years and sophomores and above, making it necessary to employ PSM to ensure comparability. All the variables had a VIF value of less than 4.

**Table 2:** Descriptive statistics of all variables

	First-year students (n = 5,908)		Sophomore and above (n = 16,101)		Differences
Variables (units)	Mean	SD	Mean	SD	t-value
BMI (kg/m^2^)	20.580	2.879	20.553	2.666	0.669
Tree (%)	3.483	7.411	2.474	4.864	11.726***
Bush (%)	2.840	2.596	2.509	2.311	9.090***
Grass (%)	26.061	15.974	23.625	15.955	10.035***
PA (hours/week)	1.521	0.601	1.590	0.627	-7.251***
Food (scores)	7.369	2.120	7.529	2.193	-4.599***
Living costs (< 1,000 / 1,000–2,000 / > 2,000 RMB)	2.448	0.755	2.540	0.778	-7.835***
Fast-food density (number/km^2^)	28.198	25.930	36.093	29.748	-18.039***
Population density (1,000 people/km^2^)	7.898	9.557	10.690	10.583	-17.789***
Street connectivity (number/km^2^)	14.914	13.451	17.542	15.312	-11.650***
Bus-stop density (number/km^2^)	4.924	3.874	5.753	4.588	-12.375***
Smoking (being or have been/never)	0.028	0.165	0.040	0.197	-4.233***
Alcohol (being or have been/never)	0.354	0.478	0.408	0.491	-7.244***
Age (year)	18.479	1.182	20.578	1.572	-93.403***
Gender (male/female)	0.439	0.496	0.446	0.497	-1.011
Type of Hukou (urban /rural)	0.591	0.492	0.611	0.488	-2.744***
Ethnicity (Han / the other)	0.860	0.348	0.868	0.338	-1.641

*, **, and *** represent the p ≤ 0.1p ≤ 0.05, p ≤ 0.01, respectively

## Effect of different types of green space on adiposity

**Fig. 2:**
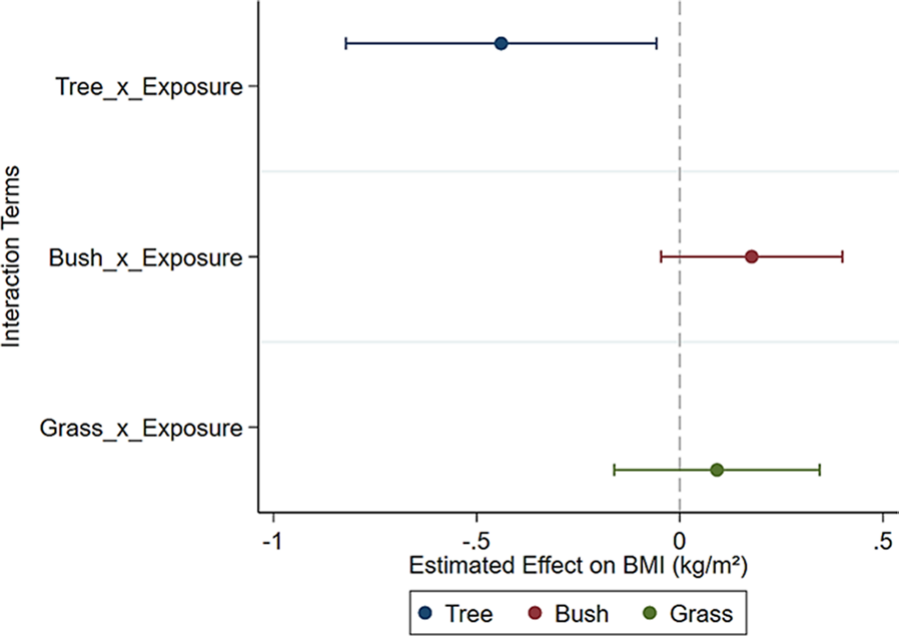
The impact of different types of green spaces on adiposity (N = 21,990)

To evaluate the causal effect of different types of green spaces on BMI, we adopted the PSM-DID) method. In Fig. 2, the confidence intervals shown in blue, red, and green represent the impact of trees, bushes, and grass on BMI, respectively. The coefficients of the interaction term $(Htree\times Exposure)$ revealed a significant negative impact of trees on BMI (Coef. = − 0.440, *p* < 0.05). There was no statistically significant association between bushes and grass and BMI. The coefficients of the control variables, year and time dummies are not reported here to save space (refer to Supplementary A for details).

## Robustness check

**Fig. 3:**
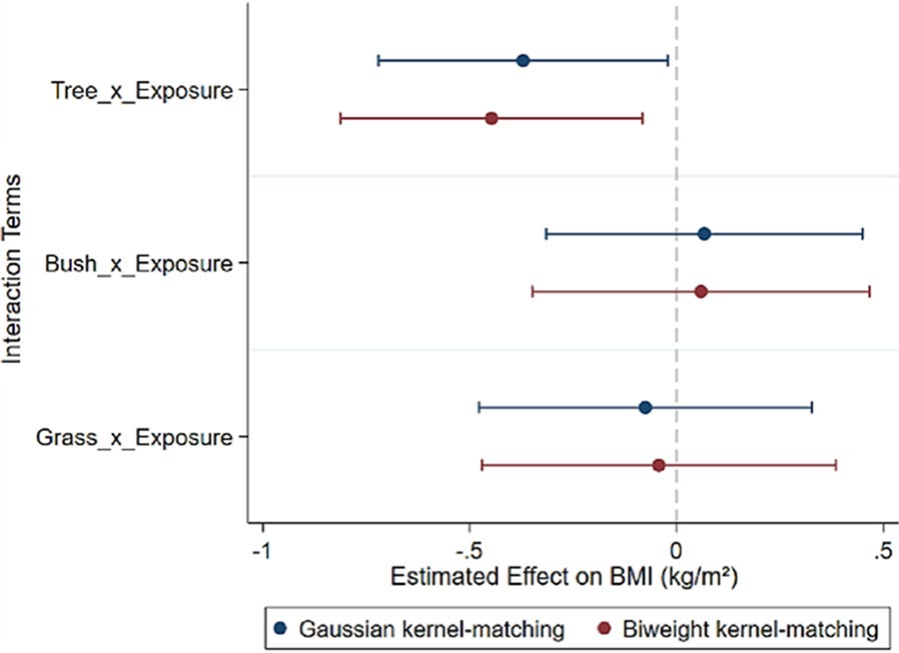
Gaussian and Biweight kernel-matching

**Fig. 4:**
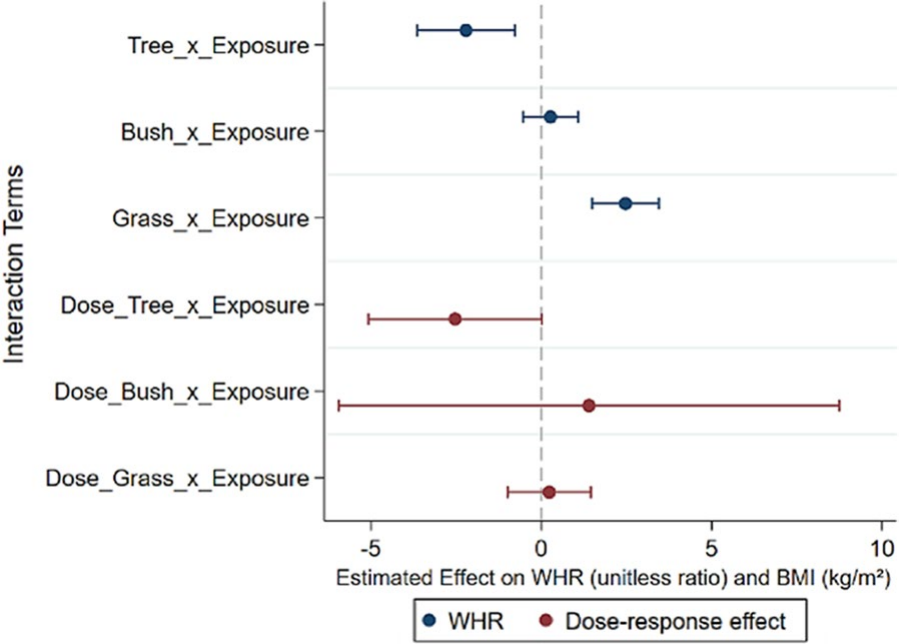
WHR and dose–response effect

To ensure the robustness of our results and mitigate any potential bias introduced by the propensity matching method, we conducted additional validation using radius matching methods. Additionally, we applied Gaussian and Biweight kernel-matching functions to match the parameters, thus reducing the bias arising from different kernel-matching functions (refer to Fig. 3 and Supplementary B1 for details). Moreover, we adopted the waist-to-hip ratio (WHR) as a measure of adiposity, following the approach of Nichani et al. [45]. Finally, we treated these variables as continuous variables, in line with the methodology of Xie et al. [71] (refer to Fig. 4 and Supplementary B2 for details). Our findings remained robust across these analyses.

To address potential concerns about omitted variable bias, we additionally controlled for overall green exposure using NDVI (Supplementary B3) and tested a model including mean-centered values of green space types (Supplementary B4). Both approaches produced results consistent with our main findings, further supporting the robustness of our conclusions.

## Mechanisms

Previous section has demonstrated the influence of green spaces on adiposity. In this section, we examined two potential mechanisms: energy intake (measured by unhealthy food intake) and energy expenditure (measured by walking duration). Following the frameworks used by Cao et al. [12] and Bianchi et al. [10], we constructed the following models:

7 $${M}_{\text{i}}={\beta }_{0}+{\beta }_{1}{Htree}_{i}+{\beta }_{2}{Exposure }_{i}+{\beta }_{1}{Htree}_{i}*{Exposure}_{i}+\sum {\tau }_{j}*{X}_{ji}+{\varepsilon }_{i}$$

8 $${M}_{\text{i}}={\beta }_{0}+{\beta }_{1}{Hbush}_{i}+{\beta }_{2}{Exposure }_{i}+{\beta }_{1}{Hbush}_{i}*{Exposure}_{i}+\sum {\tau }_{j}*{X}_{ji}+{\varepsilon }_{i}$$

9 $${M}_{\text{i}}={\beta }_{0}+{\beta }_{1}{Hgrass}_{i}+{\beta }_{2}{Exposure }_{i}+{\beta }_{1}{Hgrass}_{i}*{Exposure}_{i}+\sum {\tau }_{j}*{X}_{ji}+{\varepsilon }_{i}$$

where ${M}_{\text{i}}$ represents the potential mediators (i.e., the duration of walking and unhealthy food intake). The interaction terms ${Htree}_{i}*{Exposure}_{i}$, ${Hbush}_{i}*{Exposure}_{i}$, and ${Hgrass}_{i}*{Exposure}_{i}$ are the variables of concern, which indicates whether the undergraduates *i* influenced by tree, bush, and grass, respectively. ${\text{X}}_{\text{ji}}$ is the matrix vector of the control variables, and $\varepsilon_{i}$ is the stochastic disturbance term.

**Fig. 5:**
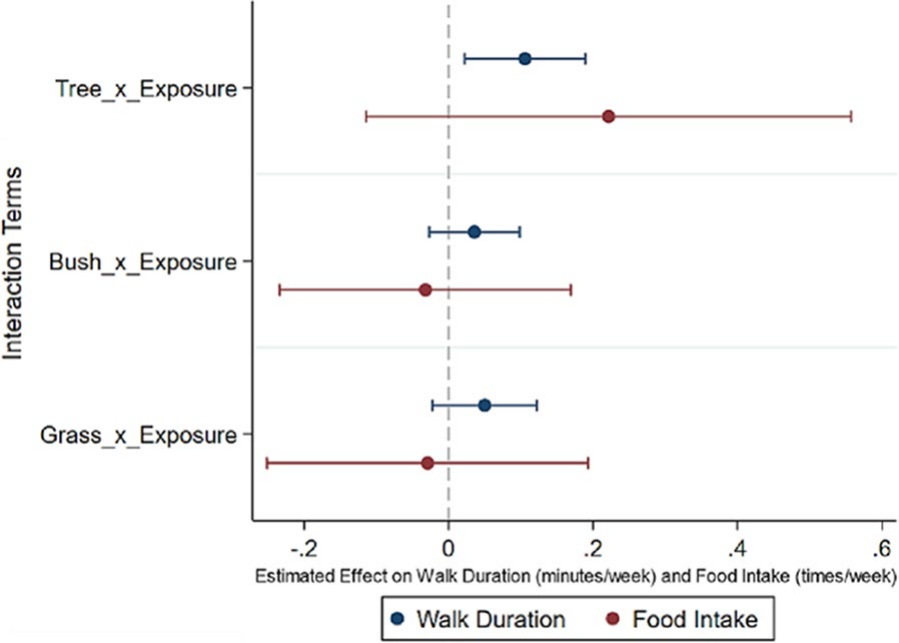
The pathways between diverse green spaces and adiposity

Figure 5 and Supplementary C depicted the impacts of trees, bushes, and grass on outdoor PA and unhealthy food intake, respectively. The findings revealed that trees had a positive impact on walking duration (Coef. = 0.106, *p* < 0.05), whereas neither bushes nor grass had a significant effect. Furthermore, the different types of green spaces did not show statistically significant impact on unhealthy food intake. These results indicated that trees could mitigate BMI by promoting walking duration.

## Heterogeneity analysis

**Fig. 6:**
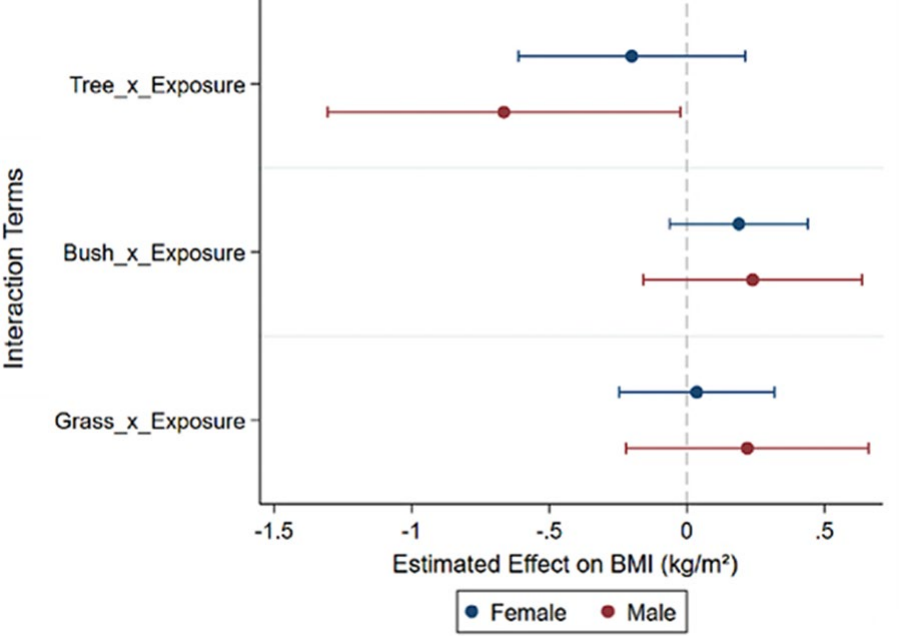
Heterogeneous effects of being male and female on adiposity

Figure 6 and Supplementary D illustrated the results for the male and female groups. The findings revealed that trees had a negative impact on the BMI of males (Coef. = -0.665, *p* < 0.05), while no statistically significant effect was observed on the BMI of females. The associations between bush and grass and BMI were found to be insignificant in both male and female groups.

## Discussion

This study adopted the PSM-DID model to estimate the causal effect of diverse types of green spaces on the adiposity status of undergraduate students in China. We also considered two mechanisms (energy expenditure and energy intake) through which green spaces may affect adiposity, as well as the heterogeneous effects on different gender groups (male vs. female). This study presents three major findings.

Our study demonstrated a plausible causal link between trees and BMI after controlling for individual and neighborhood-level variables. This finding strengthens the evidence supporting the beneficial role of trees in reducing adiposity, particularly when compared to prior cross-sectional studies. Trees, which provide shade and defined walking routes, may better support students' need for convenient outdoor spaces for PA between classes. Undergraduate students, who often face high levels of academic pressure, may find these shaded areas especially beneficial, as they offer accessible spaces for stress relief and align with their psychological and physical health needs. Additionally, given students' sensitivity to environmental aesthetics, the structure and shade of trees may offer a sense of privacy and comfort.

The mechanism analysis confirmed that trees promoted PA and thus helped prevent adiposity, while other pathways were not statistically significant. The availability of trees (mostly in open public spaces and along streets on university campuses) may encourage PA and contribute to a lower risk of adiposity [63]. Compared to grass, trees provide shaded open spaces for outdoor PA [1], making it more comfortable for students to walk or jog in parks or tree-lined streets, particularly during the summer.

However, we found no impact of bushes and grass on the adiposity of undergraduates. Grass and bushes may serve mainly decorative roles on campus and do not provide the same practical, shaded routes that can support PA in university campuses in China. Another possible reason why bushes and grass did not show significant effects on adiposity is that students may not perceive or use these green spaces as isolated elements. In real campus environments, vegetation often appears in combinations, such as grassy areas under trees or small bushes alongside paths. The health benefits of grass or bushes may depend on how they are integrated with other vegetation types. When these elements are not combined in a way that supports walking, shade, or rest, their impact on physical activity and health may be limited.

Nonetheless, other studies have documented positive associations between grass exposure and health outcomes. For instance, Astell-Burt and Feng [4] found that access to grassy areas was associated with reduced psychological distress in urban populations. Similarly, Huang et al. [29] demonstrated that grassy environments could have restorative effects in terms of reducing perceived stress. These mixed findings suggest that the health benefits of grass may vary depending on the outcome measured (e.g., mental health vs. physical health), population characteristics, or environmental settings. Our results contribute to this ongoing debate by highlighting that grass exposure, while potentially beneficial for mental wellbeing, may not have a significant impact on adiposity among undergraduates.

Regarding the pathway of energy intake, our results suggest that food intake may not play a significant role in the relationship between green spaces and adiposity. This finding contrasts with some laboratory experiments, which demonstrated immediate and transient effects of natural versus urban visual exposure on healthier food consumption within controlled settings [13]. The discrepancy may be attributed to differences between experimental and real-world settings. In laboratories, factors like time constraints, food availability, and social influences are minimized, whereas undergraduates frequently consume meals in on-campus dining facilities or nearby eateries where food choices are primarily driven by convenience and affordability rather than by environmental settings [51]. As a result, the presence of trees or green spaces may not significantly affect their eating behavior.

The heterogeneity analysis shows that trees have a significant effect on men but not on women. One plausible reason is that safety awareness differs between genders, which further influences their preferences for PA and BMI. Females are more sensitive to perceived safety [11, 19], and dense trees may reduce feelings of safety as they can potentially hide criminals. Thus, they may be less likely to engage in physical activity in tree-covered spaces. Beyond safety, behavioral differences may also play a role. Prior studies suggest that males are more likely to participate in moderate-to-vigorous physical activity in outdoor environments [52], while females tend to favor low-intensity activities such as walking, often in more structured or open spaces. Tree-covered areas, which offer shade and defined routes, may better support the types of activities preferred by males, contributing to their more pronounced reduction in adiposity. Although women may spend more time close to or in their homes, which is influenced by the residential environment, because of societal norms [32, 62], our results do not support this claim. One possible reason for this is the unique characteristics of the study sample. Unlike general female adults, female undergraduates have no household responsibilities, and hence, have similar outdoor spaces compared with those of their male counterparts.

This study has several strengths. First, we differentiated the effects of three types of green spaces on the BMI of undergraduates. These results suggest that using aggregate greenness to determine the impact of green spaces can lead to mixed results, and future studies should focus more on specific types of green spaces. Furthermore, we explored walking as a potential mediator of the effect of trees on BMI. Second, on the methodological front, we mitigated residential self-selection bias by selecting undergraduates who had limited freedom in choosing residential locations. Students and parents primarily choose a university based on its academic performance and reputation, rather than the provision of green spaces on campus. This allowed us to construct a quasi-experiment by comparing changes in the BMI of undergraduates enrolled in university campuses with different levels of green spaces. We innovatively combined the PSM and DID methods to examine causality, addressing previous knowledge gaps [20, 30, 49].

Nonetheless, some limitations of this study should be noted. First, green space exposure was estimated using a 1,000 m circular buffer based on the average campus radius, a commonly used approach in related studies. However, this static buffer assumes students are influenced only by their immediate environment and overlooks variations in daily mobility, potentially leading to exposure misclassification. This issue aligns with the Uncertain Geographic Context Problem (UGCoP), which emphasizes that environmental effects can be biased when contextual boundaries do not reflect the actual areas relevant to individuals’ experiences [33]. Future studies could address this issue by using multiple buffer sizes or incorporating mobility-aware methods, such as GPS-based tracking, to better reflect students’ dynamic exposure patterns [26].

Second, the green space data used in this study is restricted by the classification and spatial resolution of the LCZ system. While the LCZ approach supports large-scale comparative analyses, it does not capture detailed features of green space, such as vegetation density, tree species, spatial layout, or built-in facilities, which may influence health outcomes. In addition, the 30-m resolution of the LCZ data may not fully capture how students perceive and interact with mixed vegetation on campus. As green spaces often contain combinations of trees, grass, and bushes, our approach may overlook their integrated effects. Future studies could benefit from integrating higher-resolution and image-based data, such as drone-based, multispectral, or street-level imagery, to refine green space classification and capture these finer environmental characteristics.

Third, it is uncertain whether undergraduates live near the centroid of campuses and the influence of socio-political boundaries might confound the results. Future studies should take socio-political boundaries into account.

Fourth, this study does not account for potential variations in university sports class requirements due to data constraints. Future research could benefit from incorporating institution-level sports class policies when examining the impact of the built environment on student health.

## Conclusion and recommendation

Green spaces play an essential role in supporting public health. Using a quasi-experimental design and a large undergraduate sample in China, this study identified a causal link between tree and reduced adiposity, particularly among male students. Our analysis also found that increased walking mediated this relationship, highlighting the role of trees in encouraging physical activity on campus.

Campus planners should aim to balance the configuration of trees, bushes, and grass to achieve a variety of health-related outcomes. Specifically, planting trees along walkways, main pedestrian routes, and open gathering areas can create shaded, walkable environments that promote daily physical activity and reduce adiposity. At the same time, thoughtful planning is needed to ensure that tree density does not compromise visibility and perceived safety, especially for female students.

While bushes and grass did not show direct effects on adiposity in this study, previous research highlights their potential benefits for mental well-being, stress reduction, and campus aesthetics. Grassy areas can be used for leisure, relaxation, and informal social activities, while low shrubs may help define space and improve visual comfort. We recommend allocating grassy areas near dormitories and libraries for passive recreation, and using bushes to create inviting, well-structured social or quiet zones.

## Supplementary Information

Additional file 1.

## Data Availability

No datasets were generated or analysed during the current study.
