# Supplementary material for: The impacts of various green space types on the adiposity of undergraduate students: a nationwide quasi-experimental study
Source: Int J Health Geogr. 2025 Jul 17;24:15. doi: 10.1186/s12942-025-00402-0 (PMC12273302; doi:10.1186/s12942-025-00402-0)
Supplement: Supplementary file 1 — Additional file 1. [file 12942_2025_402_MOESM1_ESM.docx]

Supplementary Figure 1

Figure S1. Example of remote sensing imagery and corresponding LCZ classification map. (a) The LCZ map of Guangzhou University (Guihuagang Campus), downloaded from Geopedia (https://geopedia.world/). (b) The corresponding remote sensing image of the campus, extracted from Google Earth.


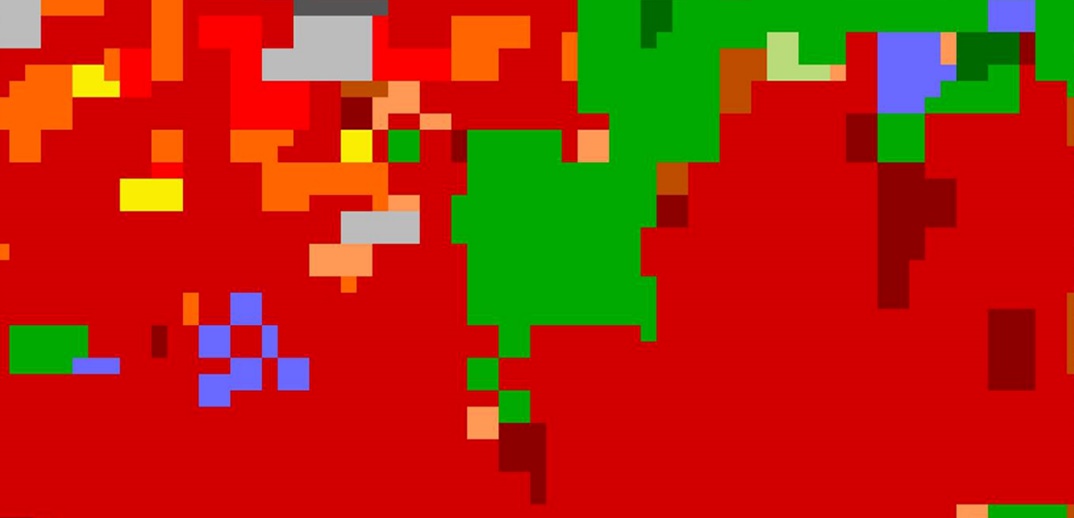

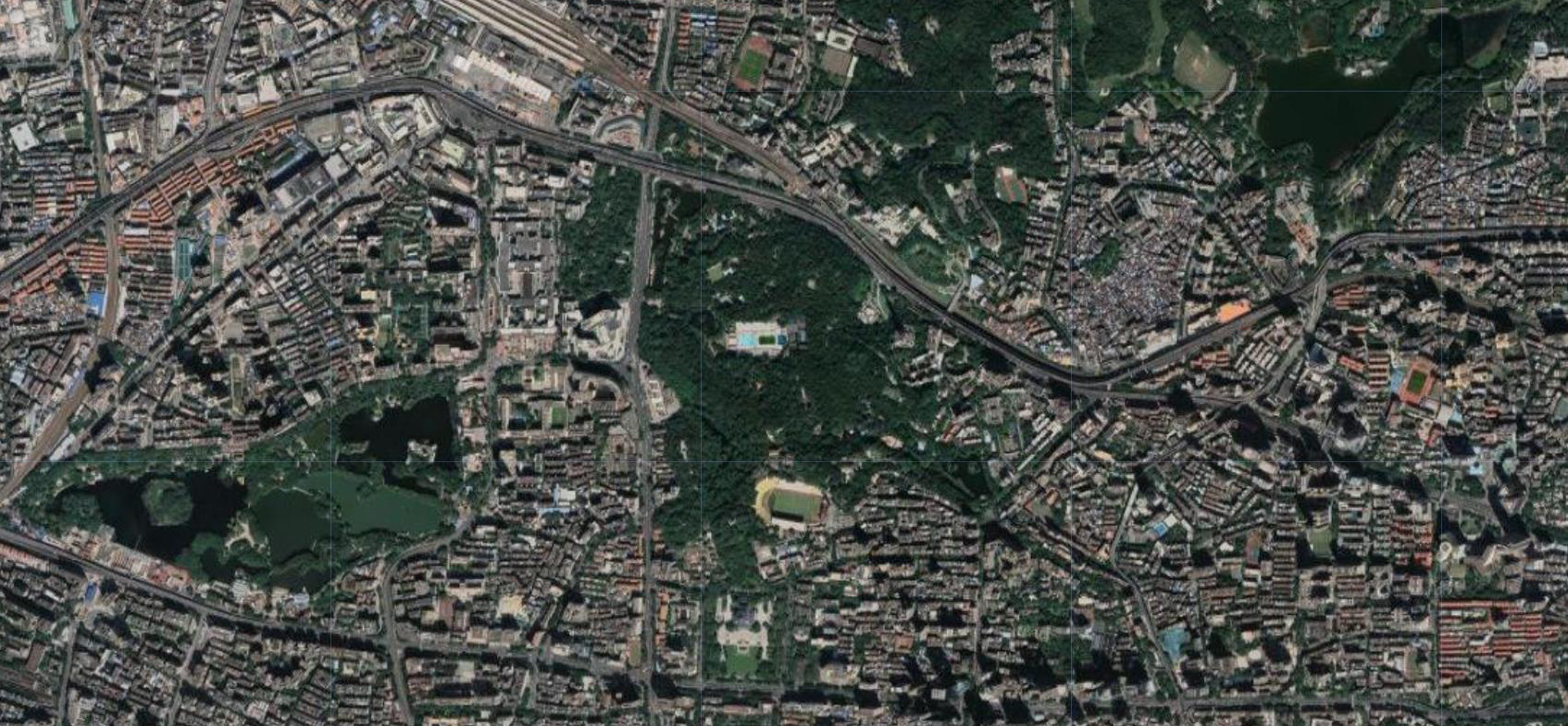


(a)

(b)

Compact high-rise

Compact mid-rise

Compact low-rise

Open high-rise

Open mid-rise

Open low-rise

Lightweight low-rise

Large low-rise

Sparsely built

Heavy industry

Dense trees

Scattered trees

Bush, shrub

Low plants

**Local Climate Zone**

Supplementary A. The impact of different types of green spaces on adiposity (N = 21,990).

|  | (1) | (2) | (3) |
| --- | --- | --- | --- |
| Variables | BMI | BMI | BMI |
|  | [95% CI] | [95% CI] | [95% CI] |
| Tree_x_Exposure | -0.440^**^ |  |  |
|  | [-0.822,-0.058] |  |  |
| Bush_x_Exposure |  | 0.177 |  |
|  |  | [-0.046,0.400] |  |
| Grass_x_Exposure |  |  | 0.092 |
|  |  |  | [-0.161,0.344] |
| HTree | 0.500^***^ |  |  |
|  | [0.147,0.852] |  |  |
| HBush |  | 0.035 |  |
|  |  | [-0.246,0.316] |  |
| HGrass |  |  | 0.005 |
|  |  |  | [-0.293,0.302] |
| Exposure | 0.138 | -0.179 | -0.134 |
|  | [-0.137,0.413] | [-0.401,0.043] | [-0.369,0.101] |
| PA | -0.004 | -0.003 | -0.003 |
|  | [-0.029,0.021] | [-0.028,0.022] | [-0.028,0.022] |
| Living costs | 0.037 | -0.002 | 0.001 |
|  | [-0.189,0.264] | [-0.226,0.222] | [-0.225,0.228] |
| Fast-food density | -0.002 | -0.002 | -0.001 |
|  | [-0.007,0.004] | [-0.008,0.003] | [-0.007,0.004] |
| Population density | 0.006 | 0.001 | 0.006 |
|  | [-0.013,0.025] | [-0.019,0.020] | [-0.013,0.024] |
| Street connectivity | 0.001 | 0.004 | 0.003 |
|  | [-0.013,0.015] | [-0.010,0.019] | [-0.012,0.017] |
| Bus-stop density | 0.027 | 0.019 | 0.028 |
|  | [-0.022,0.076] | [-0.029,0.068] | [-0.023,0.079] |
| Smoking | 0.276 | 0.303 | 0.338 |
|  | [-0.643,1.196] | [-0.615,1.221] | [-0.576,1.251] |
| Alcohol | 0.057 | 0.058 | 0.050 |
|  | [-0.233,0.347] | [-0.232,0.348] | [-0.240,0.339] |
| Age | 0.011 | 0.017 | 0.019 |
|  | [-0.080,0.103] | [-0.076,0.110] | [-0.073,0.112] |
| Gender | 1.667^***^ | 1.676^***^ | 1.668^***^ |
|  | [1.398,1.935] | [1.408,1.943] | [1.401,1.936] |
| Type of Hukou | 0.209 | 0.182 | 0.162 |
|  | [-0.048,0.466] | [-0.079,0.442] | [-0.099,0.423] |
| Ethnicity | 0.046 | 0.041 | -0.005 |
|  | [-0.341,0.433] | [-0.337,0.419] | [-0.387,0.376] |
| Tree |  | -0.532 | -0.251 |
|  |  | [-1.948,0.885] | [-1.667,1.165] |
| Bush | -3.796 |  | 1.244 |
|  | [-9.940,2.348] |  | [-4.457,6.945] |
| Grass | -0.957^**^ | -0.870^*^ |  |
|  | [-1.899,-0.015] | [-1.755,0.016] |  |
| Constant | 19.301^***^ | 19.451^***^ | 19.099^***^ |
|  | [17.429,21.174] | [17.502,21.400] | [17.236,20.963] |
| F | 11.723 | 11.129 | 11.015 |
| R2 | 0.107 | 0.103 | 0.101 |

Note: *, **, and *** represent the p≤0.01, p≤0.05, p≤0.1, respectively. Units: Beta and [95% CI].

Supplementary B1. Robust checks: The impact of different types of green spaces on adiposity.

|  | (1) | (2) | (3) | (4) | (5) | (6) |
| --- | --- | --- | --- | --- | --- | --- |
|  | Gaussian kernel-matching | | | Biweight kernel-matching | | |
| Variables | BMI | BMI | BMI | BMI | BMI | BMI |
|  | [95% CI] | [95% CI] | [95% CI] | [95% CI] | [95% CI] | [95% CI] |
| Tree_x_Exposure | -0.371^**^ |  |  | -0.447^**^ |  |  |
|  | [-0.737,-0.004] |  |  | [-0.830,-0.064] |  |  |
| Bush_x_Exposure |  | 0.194^*^ |  |  | 0.167 |  |
|  |  | [-0.019,0.408] |  |  | [-0.057,0.391] |  |
| Grass_x_Exposure |  |  | 0.082 |  |  | 0.091 |
|  |  |  | [-0.160,0.324] |  |  | [-0.162,0.345] |
| HTree | 0.462^***^ |  |  | 0.497^***^ |  |  |
|  | [0.127,0.798] |  |  | [0.144,0.851] |  |  |
| HBush |  | 0.018 |  |  | 0.040 |  |
|  |  | [-0.248,0.284] |  |  | [-0.243,0.323] |  |
| HGrass |  |  | -0.013 |  |  | 0.002 |
|  |  |  | [-0.293,0.268] |  |  | [-0.297,0.302] |
| Exposure | 0.092 | -0.196^*^ | -0.139 | 0.142 | -0.174 | -0.133 |
|  | [-0.169,0.352] | [-0.408,0.015] | [-0.363,0.086] | [-0.133,0.418] | [-0.396,0.048] | [-0.369,0.102] |
| PA | -0.005 | -0.004 | -0.004 | -0.004 | -0.003 | -0.003 |
|  | [-0.028,0.019] | [-0.028,0.020] | [-0.027,0.020] | [-0.029,0.021] | [-0.029,0.022] | [-0.028,0.022] |
| Living costs | 0.026 | -0.013 | -0.009 | 0.033 | -0.005 | -0.002 |
|  | [-0.186,0.239] | [-0.223,0.197] | [-0.222,0.204] | [-0.194,0.260] | [-0.230,0.220] | [-0.230,0.225] |
| Fast-food density | -0.002 | -0.003 | -0.001 | -0.002 | -0.002 | -0.001 |
|  | [-0.007,0.003] | [-0.008,0.003] | [-0.007,0.004] | [-0.007,0.004] | [-0.008,0.003] | [-0.007,0.004] |
| Population density | 0.005 | 0.001 | 0.006 | 0.006 | 0.001 | 0.006 |
|  | [-0.013,0.024] | [-0.017,0.020] | [-0.012,0.023] | [-0.013,0.026] | [-0.019,0.021] | [-0.013,0.025] |
| Street connectivity | 0.001 | 0.004 | 0.002 | 0.001 | 0.004 | 0.003 |
|  | [-0.012,0.014] | [-0.011,0.018] | [-0.011,0.016] | [-0.013,0.014] | [-0.010,0.019] | [-0.012,0.017] |
| Bus-stop density | 0.026 | 0.020 | 0.028 | 0.027 | 0.019 | 0.028 |
|  | [-0.021,0.074] | [-0.027,0.067] | [-0.021,0.078] | [-0.022,0.076] | [-0.029,0.068] | [-0.023,0.079] |
| Smoking | 0.241 | 0.264 | 0.298 | 0.280 | 0.307 | 0.342 |
|  | [-0.621,1.103] | [-0.596,1.125] | [-0.560,1.155] | [-0.642,1.202] | [-0.613,1.227] | [-0.574,1.258] |
| Alcohol | 0.054 | 0.053 | 0.044 | 0.060 | 0.060 | 0.052 |
|  | [-0.222,0.330] | [-0.222,0.328] | [-0.231,0.319] | [-0.231,0.351] | [-0.231,0.350] | [-0.238,0.342] |
| Age | 0.015 | 0.020 | 0.023 | 0.011 | 0.016 | 0.019 |
|  | [-0.074,0.104] | [-0.071,0.111] | [-0.067,0.113] | [-0.080,0.102] | [-0.077,0.109] | [-0.074,0.111] |
| Gender | 1.628^***^ | 1.637^***^ | 1.630^***^ | 1.666^***^ | 1.676^***^ | 1.668^***^ |
|  | [1.372,1.884] | [1.383,1.892] | [1.376,1.884] | [1.397,1.936] | [1.407,1.944] | [1.400,1.936] |
| Type of Hukou | 0.226^*^ | 0.199 | 0.181 | 0.210 | 0.183 | 0.163 |
|  | [-0.017,0.468] | [-0.046,0.445] | [-0.064,0.427] | [-0.048,0.469] | [-0.079,0.445] | [-0.099,0.425] |
| Ethnicity | 0.058 | 0.049 | 0.007 | 0.050 | 0.046 | 0.000 |
|  | [-0.312,0.428] | [-0.312,0.410] | [-0.357,0.371] | [-0.337,0.438] | [-0.332,0.425] | [-0.381,0.382] |
| Tree |  | -0.395 | -0.123 |  | -0.544 | -0.262 |
|  |  | [-1.691,0.901] | [-1.413,1.167] |  | [-1.974,0.887] | [-1.693,1.169] |
| Bush | -3.581 |  | 1.234 | -3.667 |  | 1.346 |
|  | [-9.348,2.186] |  | [-4.200,6.668] | [-9.846,2.512] |  | [-4.390,7.081] |
| Grass | -0.971^**^ | -0.890^**^ |  | -0.960^**^ | -0.879^*^ |  |
|  | [-1.852,-0.091] | [-1.716,-0.065] |  | [-1.908,-0.012] | [-1.771,0.012] |  |
| Constant | 19.286^***^ | 19.437^***^ | 19.079^***^ | 19.315^***^ | 19.466^***^ | 19.112^***^ |
|  | [17.460,21.112] | [17.545,21.329] | [17.262,20.895] | [17.448,21.182] | [17.521,21.410] | [17.253,20.971] |
| F | 12.137 | 11.647 | 11.476 | 11.687 | 11.066 | 10.971 |
| R2 | 0.102 | 0.099 | 0.097 | 0.107 | 0.103 | 0.101 |

Note: *, **, and *** represent the p≤0.01, p≤0.05, p≤0.1, respectively. Units: Beta and [95% CI].

Supplementary B2. Robust checks: The impact of different types of green spaces on adiposity.

|  | (1) | (2) | (3) | (4) | (5) | (6) |
| --- | --- | --- | --- | --- | --- | --- |
|  | PSM-DID | | | Dose-response effect | | |
| Variables | WHR | WHR | WHR | BMI | BMI | BMI |
|  | [95% CI] | [95% CI] | [95% CI] | [95% CI] | [95% CI] | [95% CI] |
| Tree_x_Exposure | -2.197^***^ |  |  |  |  |  |
|  | [-3.632,-0.763] |  |  |  |  |  |
| Bush_x_Exposure |  | 0.301 |  |  |  |  |
|  |  | [-0.505,1.107] |  |  |  |  |
| Grass_x_Exposure |  |  | 2.489^***^ |  |  |  |
|  |  |  | [1.516,3.462] |  |  |  |
| Dose_Tree_x_Exposure |  |  |  | -2.528^*^ |  |  |
|  |  |  |  | [-5.056,0.000] |  |  |
| Dose_Bush_x_Exposure |  |  |  |  | 1.609 |  |
|  |  |  |  |  | [-5.703,8.921] |  |
| Dose_Grass_x_Exposure |  |  |  |  |  | 0.211 |
|  |  |  |  |  |  | [-1.007,1.430] |
| HTree | 3.112^***^ |  |  |  |  |  |
|  | [1.729,4.495] |  |  |  |  |  |
| HBush |  | 1.918^***^ |  |  |  |  |
|  |  | [0.957,2.880] |  |  |  |  |
| HGrass |  |  | 0.817 |  |  |  |
|  |  |  | [-0.315,1.950] |  |  |  |
| Exposure | 0.862 | -0.471 | -1.509^***^ | -0.001 | -0.122 | -0.136 |
|  | [-0.201,1.926] | [-1.321,0.379] | [-2.407,-0.610] | [-0.213,0.210] | [-0.404,0.159] | [-0.490,0.217] |
| PA | -0.078 | -0.079 | -0.076 | -0.005 | -0.003 | -0.004 |
|  | [-0.188,0.031] | [-0.192,0.034] | [-0.189,0.037] | [-0.030,0.021] | [-0.029,0.022] | [-0.029,0.022] |
| Living costs | -0.568 | -0.704 | -0.819^*^ | -0.001 | -0.004 | -0.003 |
|  | [-1.459,0.322] | [-1.578,0.170] | [-1.695,0.057] | [-0.227,0.226] | [-0.230,0.222] | [-0.229,0.223] |
| Fast-food density | -0.005 | -0.008 | -0.014 | -0.003 | -0.002 | -0.002 |
|  | [-0.024,0.013] | [-0.027,0.010] | [-0.032,0.004] | [-0.008,0.003] | [-0.008,0.003] | [-0.008,0.003] |
| Population density | -0.038 | -0.071^**^ | -0.078^**^ | 0.003 | 0.001 | 0.001 |
|  | [-0.098,0.021] | [-0.134,-0.009] | [-0.137,-0.018] | [-0.017,0.023] | [-0.019,0.021] | [-0.019,0.021] |
| Street connectivity | -0.098^***^ | -0.058^**^ | -0.076^***^ | 0.003 | 0.003 | 0.004 |
|  | [-0.151,-0.044] | [-0.116,-0.000] | [-0.133,-0.019] | [-0.011,0.017] | [-0.011,0.018] | [-0.011,0.018] |
| Bus-stop density | 0.391^***^ | 0.349^***^ | 0.287^***^ | 0.014 | 0.015 | 0.014 |
|  | [0.225,0.558] | [0.182,0.516] | [0.107,0.466] | [-0.036,0.063] | [-0.035,0.064] | [-0.036,0.064] |
| Smoking | -2.737^*^ | -2.595^*^ | -2.646^*^ | 0.304 | 0.300 | 0.297 |
|  | [-5.502,0.027] | [-5.247,0.057] | [-5.318,0.027] | [-0.613,1.221] | [-0.616,1.215] | [-0.618,1.213] |
| Alcohol | -0.082 | -0.154 | -0.120 | 0.059 | 0.054 | 0.054 |
|  | [-1.096,0.932] | [-1.178,0.870] | [-1.141,0.901] | [-0.231,0.349] | [-0.236,0.344] | [-0.237,0.344] |
| Age | 0.287 | 0.318 | 0.309 | 0.019 | 0.018 | 0.018 |
|  | [-0.105,0.679] | [-0.093,0.729] | [-0.108,0.725] | [-0.075,0.112] | [-0.076,0.111] | [-0.075,0.112] |
| Gender | 5.650^***^ | 5.683^***^ | 5.803^***^ | 1.675^***^ | 1.680^***^ | 1.681^***^ |
|  | [4.661,6.640] | [4.693,6.673] | [4.805,6.800] | [1.407,1.944] | [1.412,1.949] | [1.412,1.950] |
| Type of Hukou | 1.858^***^ | 1.743^***^ | 1.722^***^ | 0.185 | 0.181 | 0.180 |
|  | [0.962,2.753] | [0.848,2.638] | [0.823,2.621] | [-0.075,0.445] | [-0.079,0.441] | [-0.080,0.440] |
| Ethnicity | 1.048 | 1.099 | 0.906 | 0.029 | 0.033 | 0.031 |
|  | [-0.368,2.463] | [-0.319,2.518] | [-0.534,2.347] | [-0.352,0.410] | [-0.348,0.414] | [-0.350,0.412] |
| Tree |  | -4.780^*^ | -4.432 | 0.274 | -0.410 | -0.444 |
|  |  | [-10.235,0.675] | [-10.201,1.338] | [-1.435,1.982] | [-1.796,0.977] | [-1.839,0.951] |
| Bush | -13.211 |  | -10.226 | -1.371 | -1.678 | -1.262 |
|  | [-35.344,8.922] |  | [-32.906,12.454] | [-7.046,4.305] | [-8.542,5.185] | [-6.975,4.450] |
| Grass | 4.125^**^ | 5.450^***^ |  | -0.970^**^ | -1.007^**^ | -1.077^*^ |
|  | [0.858,7.393] | [2.392,8.507] |  | [-1.922,-0.018] | [-1.957,-0.058] | [-2.307,0.153] |
| Constant | 70.961^***^ | 70.745^***^ | 74.137^***^ | 19.516^***^ | 19.580^***^ | 19.573^***^ |
|  | [62.500,79.421] | [62.034,79.456] | [65.653,82.622] | [17.599,21.432] | [17.652,21.507] | [17.650,21.496] |
| N | 20160 | 20160 | 20160 | 21990 | 21990 | 21990 |
| F | 17.161 | 17.615 | 18.441 | 11.534 | 11.003 | 11.413 |
| R2 | 0.124 | 0.117 | 0.113 | 0.103 | 0.103 | 0.103 |

Note: *, **, and *** represent the p≤0.01, p≤0.05, p≤0.1, respectively. Units: Beta and [95% CI].

Supplementary B3. Robust checks: The impact of different types of green spaces on adiposity using NDVI as a composite control variable.

|  | (1) | (2) | (3) |
| --- | --- | --- | --- |
| Variables | BMI | BMI | BMI |
|  | [95% CI] | [95% CI] | [95% CI] |
| Tree_x_Exposure | -0.348^*^ |  |  |
|  | [-0.727,0.032] |  |  |
| Bush_x_Exposure |  | 0.163 |  |
|  |  | [-0.060,0.386] |  |
| Grass_x_Exposure |  |  | 0.127 |
|  |  |  | [-0.094,0.348] |
| HTree | 0.574^***^ |  |  |
|  | [0.214,0.934] |  |  |
| HBush |  | 0.068 |  |
|  |  | [-0.208,0.345] |  |
| HGrass |  |  | 0.121 |
|  |  |  | [-0.174,0.415] |
| Exposure | 0.091 | -0.169 | -0.145 |
|  | [-0.182,0.364] | [-0.391,0.052] | [-0.365,0.076] |
| NDVI | -3.124^***^ | -2.450^***^ | -2.761^***^ |
|  | [-4.805,-1.443] | [-3.976,-0.925] | [-4.292,-1.230] |
| PA | -0.006 | -0.005 | -0.005 |
|  | [-0.031,0.020] | [-0.031,0.021] | [-0.030,0.020] |
| Living costs | 0.023 | -0.014 | -0.016 |
|  | [-0.201,0.247] | [-0.238,0.209] | [-0.240,0.209] |
| Fast-food density | -0.002 | -0.002 | -0.002 |
|  | [-0.007,0.004] | [-0.008,0.003] | [-0.008,0.003] |
| Population density | 0.007 | 0.002 | 0.004 |
|  | [-0.013,0.026] | [-0.017,0.022] | [-0.016,0.023] |
| Street connectivity | 0.003 | 0.005 | 0.003 |
|  | [-0.012,0.017] | [-0.010,0.019] | [-0.011,0.018] |
| Bus-stop density | 0.027 | 0.017 | 0.019 |
|  | [-0.022,0.076] | [-0.031,0.065] | [-0.031,0.070] |
| Smoking | 0.269 | 0.312 | 0.322 |
|  | [-0.651,1.188] | [-0.608,1.231] | [-0.589,1.234] |
| Alcohol | 0.068 | 0.061 | 0.055 |
|  | [-0.222,0.359] | [-0.230,0.352] | [-0.235,0.346] |
| Age | 0.003 | 0.013 | 0.012 |
|  | [-0.087,0.093] | [-0.079,0.105] | [-0.079,0.104] |
| Gender | 1.648^***^ | 1.663^***^ | 1.668^***^ |
|  | [1.379,1.918] | [1.395,1.931] | [1.399,1.936] |
| Type of Hukou | 0.227^*^ | 0.189 | 0.182 |
|  | [-0.031,0.485] | [-0.074,0.452] | [-0.080,0.444] |
| Ethnicity | 0.074 | 0.031 | -0.006 |
|  | [-0.311,0.460] | [-0.346,0.407] | [-0.383,0.372] |
| Constant | 19.860^***^ | 19.928^***^ | 20.025^***^ |
|  | [17.991,21.729] | [17.996,21.860] | [18.129,21.922] |
| N | 21990 | 21990 | 21990 |
| F | 12.660 | 11.830 | 11.829 |
| R2 | 0.110 | 0.104 | 0.104 |

Note: *, **, and *** represent the p≤0.01, p≤0.05, p≤0.1, respectively.

Supplementary B4. Robust checks: The impact of different types of green spaces on adiposity using mean-centered values.

|  | (1) | (2) | (3) |
| --- | --- | --- | --- |
| Variables | BMI | BMI | BMI |
|  | [95% CI] | [95% CI] | [95% CI] |
| CTree_x_Exposure | -0.432^**^ |  |  |
|  | [-0.812,-0.052] |  |  |
| CBush_x_Exposure |  | 0.062 |  |
|  |  | [-0.329,0.454] |  |
| CGrass_x_Exposure |  |  | -0.049 |
|  |  |  | [-0.443,0.345] |
| HTree | 0.497^***^ |  |  |
|  | [0.146,0.848] |  |  |
| HBush |  | 0.019 |  |
|  |  | [-0.355,0.393] |  |
| HGrass |  |  | 0.023 |
|  |  |  | [-0.360,0.407] |
| Exposure | 0.135 | -0.116 | -0.058 |
|  | [-0.140,0.411] | [-0.391,0.159] | [-0.331,0.215] |
| PA | -0.004 | -0.003 | -0.003 |
|  | [-0.029,0.021] | [-0.029,0.022] | [-0.028,0.022] |
| Living costs | 0.037 | -0.002 | 0.001 |
|  | [-0.189,0.264] | [-0.226,0.221] | [-0.225,0.228] |
| Fast-food density | -0.002 | -0.002 | -0.001 |
|  | [-0.007,0.004] | [-0.008,0.003] | [-0.007,0.004] |
| Population density | 0.006 | 0.001 | 0.006 |
|  | [-0.014,0.025] | [-0.018,0.021] | [-0.013,0.024] |
| Street connectivity | 0.001 | 0.004 | 0.002 |
|  | [-0.013,0.015] | [-0.011,0.019] | [-0.012,0.017] |
| Bus-stop density | 0.027 | 0.017 | 0.030 |
|  | [-0.022,0.076] | [-0.031,0.065] | [-0.022,0.081] |
| Smoking | 0.276 | 0.301 | 0.336 |
|  | [-0.643,1.196] | [-0.617,1.220] | [-0.577,1.249] |
| Alcohol | 0.058 | 0.054 | 0.050 |
|  | [-0.233,0.348] | [-0.235,0.343] | [-0.240,0.339] |
| Age | 0.012 | 0.017 | 0.019 |
|  | [-0.080,0.103] | [-0.076,0.110] | [-0.073,0.112] |
| Gender | 1.666^***^ | 1.678^***^ | 1.668^***^ |
|  | [1.398,1.935] | [1.410,1.945] | [1.401,1.935] |
| Type of Hukou | 0.209 | 0.181 | 0.162 |
|  | [-0.048,0.466] | [-0.080,0.442] | [-0.098,0.422] |
| Ethnicity | 0.046 | 0.042 | -0.005 |
|  | [-0.341,0.433] | [-0.336,0.421] | [-0.387,0.376] |
| Tree |  | -0.519 | -0.272 |
|  |  | [-1.936,0.899] | [-1.684,1.139] |
| Bush | -3.788 |  | 1.638 |
|  | [-9.932,2.355] |  | [-3.649,6.924] |
| Grass | -0.958^**^ | -0.906^**^ |  |
|  | [-1.900,-0.016] | [-1.795,-0.017] |  |
| Constant | 19.296^***^ | 19.470^***^ | 19.080^***^ |
|  | [17.424,21.169] | [17.504,21.437] | [17.213,20.947] |
| N | 21990 | 21990 | 21990 |
| F | 11.721 | 10.996 | 11.241 |
| r2_a | 0.107 | 0.103 | 0.101 |

Note: *, **, and *** represent the p≤0.01, p≤0.05, p≤0.1, respectively.

Supplementary C. The pathways between diverse green spaces and adiposity

|  | (1) | (2) | (3) | (4) | (5) | (6) |
| --- | --- | --- | --- | --- | --- | --- |
|  | Energy intake | | | Energy expenditure | | |
| Variables | Duration of walking | Duration of walking | Duration of walking | Unhealthy food intake | Unhealthy food intake | Unhealthy food intake |
|  | [95% CI] | [95% CI] | [95% CI] | [95% CI] | [95% CI] | [95% CI] |
| Tree_x_Exposure | 0.106^**^ |  |  | 0.221 |  |  |
|  | [0.022,0.189] |  |  | [-0.114,0.557] |  |  |
| Bush_x_Exposure |  | 0.036 |  |  | -0.032 |  |
|  |  | [-0.027,0.098] |  |  | [-0.234,0.170] |  |
| Grass_x_Exposure |  |  | 0.050 |  |  | -0.029 |
|  |  |  | [-0.022,0.122] |  |  | [-0.251,0.193] |
| HTree | -0.012 |  |  | -0.347^**^ |  |  |
|  | [-0.078,0.055] |  |  | [-0.646,-0.049] |  |  |
| HBush |  | -0.041 |  |  | -0.066 |  |
|  |  | [-0.092,0.009] |  |  | [-0.286,0.154] |  |
| HGrass |  |  | -0.005 |  |  | 0.090 |
|  |  |  | [-0.077,0.068] |  |  | [-0.133,0.313] |
| Exposure | -0.105^***^ | -0.062^**^ | -0.074^**^ | 0.088 | 0.217^**^ | 0.213^**^ |
|  | [-0.170,-0.041] | [-0.113,-0.011] | [-0.131,-0.017] | [-0.145,0.321] | [0.030,0.403] | [0.017,0.408] |
| PA | 0.990^***^ | 0.990^***^ | 0.990^***^ | 0.022^*^ | 0.022^*^ | 0.021^*^ |
|  | [0.983,0.997] | [0.983,0.997] | [0.983,0.997] | [-0.001,0.045] | [-0.002,0.045] | [-0.002,0.044] |
| Living costs | -0.004 | -0.008 | -0.004 | 0.383^***^ | 0.414^***^ | 0.418^***^ |
|  | [-0.038,0.029] | [-0.041,0.025] | [-0.036,0.029] | [0.188,0.577] | [0.220,0.608] | [0.224,0.613] |
| Fast-food density | -0.000 | 0.000 | 0.000 | -0.002 | -0.001 | -0.001 |
|  | [-0.001,0.001] | [-0.001,0.001] | [-0.001,0.001] | [-0.006,0.003] | [-0.005,0.004] | [-0.006,0.003] |
| Population density | 0.001 | 0.002 | 0.002^*^ | 0.022^***^ | 0.025^***^ | 0.023^***^ |
|  | [-0.001,0.004] | [-0.001,0.004] | [-0.000,0.005] | [0.007,0.037] | [0.010,0.041] | [0.009,0.038] |
| Street connectivity | 0.003^**^ | 0.003^*^ | 0.003^*^ | 0.003 | -0.001 | -0.000 |
|  | [0.000,0.006] | [-0.000,0.006] | [-0.000,0.006] | [-0.008,0.014] | [-0.013,0.011] | [-0.012,0.011] |
| Bus-stop density | -0.012^**^ | -0.010^*^ | -0.010 | 0.009 | 0.019 | 0.019 |
|  | [-0.022,-0.002] | [-0.021,0.001] | [-0.022,0.002] | [-0.029,0.048] | [-0.019,0.057] | [-0.020,0.058] |
| Smoking | 0.032 | 0.035 | 0.038 | 0.255 | 0.242 | 0.234 |
|  | [-0.086,0.150] | [-0.086,0.156] | [-0.081,0.157] | [-0.386,0.896] | [-0.401,0.885] | [-0.411,0.879] |
| Alcohol | 0.018 | 0.019 | 0.017 | -0.002 | 0.002 | 0.001 |
|  | [-0.027,0.063] | [-0.026,0.065] | [-0.029,0.063] | [-0.242,0.238] | [-0.239,0.242] | [-0.239,0.241] |
| Age | 0.012^*^ | 0.012 | 0.012 | -0.036 | -0.038 | -0.041 |
|  | [-0.002,0.026] | [-0.003,0.026] | [-0.003,0.027] | [-0.110,0.038] | [-0.112,0.036] | [-0.115,0.034] |
| Gender | -0.054^**^ | -0.054^**^ | -0.055^**^ | -0.420^***^ | -0.431^***^ | -0.427^***^ |
|  | [-0.096,-0.011] | [-0.096,-0.011] | [-0.097,-0.012] | [-0.660,-0.181] | [-0.671,-0.191] | [-0.667,-0.188] |
| Type of Hukou | 0.036 | 0.035 | 0.035 | -0.062 | -0.040 | -0.033 |
|  | [-0.015,0.086] | [-0.013,0.084] | [-0.014,0.084] | [-0.274,0.151] | [-0.253,0.172] | [-0.246,0.181] |
| Ethnicity | -0.060^**^ | -0.059^**^ | -0.063^**^ | -0.142 | -0.142 | -0.133 |
|  | [-0.110,-0.010] | [-0.108,-0.009] | [-0.115,-0.011] | [-0.426,0.142] | [-0.428,0.144] | [-0.422,0.156] |
| Tree |  | 0.247^**^ | 0.315^**^ |  | 1.197^*^ | 0.983 |
|  |  | [0.010,0.484] | [0.040,0.590] |  | [-0.016,2.410] | [-0.247,2.213] |
| Bush | -0.799 |  | -0.718 | 2.792 |  | -0.604 |
|  | [-2.380,0.781] |  | [-2.204,0.769] | [-1.527,7.112] |  | [-4.927,3.720] |
| Grass | -0.133 | -0.099 |  | 0.505 | 0.477 |  |
|  | [-0.345,0.080] | [-0.296,0.097] |  | [-0.190,1.200] | [-0.191,1.145] |  |
| Constant | -0.219 | -0.243 | -0.294^*^ | 7.404^***^ | 7.248^***^ | 7.360^***^ |
|  | [-0.521,0.084] | [-0.548,0.062] | [-0.599,0.011] | [5.859,8.949] | [5.677,8.818] | [5.838,8.883] |
| N | 21990 | 21990 | 21990 | 19691 | 19691 | 19691 |
| F | 6125.264 | 6415.062 | 6063.092 | 5.621 | 5.347 | 5.312 |
| R2 | 0.981 | 0.980 | 0.980 | 0.054 | 0.052 | 0.051 |

Note: *, **, and *** represent the p≤0.01, p≤0.05, p≤0.1, respectively. Units: Beta and [95% CI].

Supplementary D. Heterogeneous effect of being male and female on adiposity.

|  | (1) | (2) | (3) | (4) | (5) | (6) |
| --- | --- | --- | --- | --- | --- | --- |
|  | Female | | | Male | | |
| Variables | BMI | BMI | BMI | BMI | BMI | BMI |
|  | [95% CI] | [95% CI] | [95% CI] | [95% CI] | [95% CI] | [95% CI] |
| Tree_x_Exposure | -0.200 |  |  | -0.665^**^ |  |  |
|  | [-0.613,0.212] |  |  | [-1.306,-0.025] |  |  |
| Bush_x_Exposure |  | 0.189 |  |  | 0.239 |  |
|  |  | [-0.062,0.439] |  |  | [-0.159,0.636] |  |
| Grass_x_Exposure |  |  | 0.036 |  |  | 0.219 |
|  |  |  | [-0.246,0.317] |  |  | [-0.221,0.660] |
| HTree | 0.125 |  |  | 0.865^***^ |  |  |
|  | [-0.244,0.494] |  |  | [0.278,1.453] |  |  |
| HBush |  | -0.036 |  |  | 0.153 |  |
|  |  | [-0.316,0.244] |  |  | [-0.325,0.632] |  |
| HGrass |  |  | -0.073 |  |  | 0.041 |
|  |  |  | [-0.367,0.221] |  |  | [-0.469,0.550] |
| Exposure | -0.003 | -0.197 | -0.119 | 0.263 | -0.219 | -0.214 |
|  | [-0.296,0.290] | [-0.435,0.040] | [-0.378,0.139] | [-0.217,0.744] | [-0.606,0.169] | [-0.613,0.184] |
| PA | 0.020 | 0.020 | 0.019 | -0.026 | -0.024 | -0.022 |
|  | [-0.015,0.055] | [-0.015,0.055] | [-0.016,0.054] | [-0.062,0.009] | [-0.060,0.013] | [-0.058,0.014] |
| Living costs | -0.084 | -0.087 | -0.083 | 0.178 | 0.114 | 0.119 |
|  | [-0.318,0.149] | [-0.321,0.147] | [-0.315,0.148] | [-0.206,0.561] | [-0.264,0.491] | [-0.263,0.502] |
| Fast-food density | -0.003 | -0.002 | -0.002 | 0.000 | -0.001 | 0.001 |
|  | [-0.008,0.003] | [-0.008,0.003] | [-0.008,0.004] | [-0.009,0.010] | [-0.010,0.009] | [-0.008,0.011] |
| Population density | 0.027^**^ | 0.026^**^ | 0.027^**^ | -0.015 | -0.025 | -0.018 |
|  | [0.005,0.049] | [0.004,0.048] | [0.005,0.049] | [-0.046,0.016] | [-0.056,0.007] | [-0.048,0.013] |
| Street connectivity | -0.010 | -0.010 | -0.010 | 0.014 | 0.026^**^ | 0.023^*^ |
|  | [-0.025,0.006] | [-0.026,0.006] | [-0.026,0.005] | [-0.010,0.037] | [0.002,0.051] | [-0.001,0.047] |
| Bus-stop density | 0.017 | 0.018 | 0.016 | 0.031 | -0.002 | 0.020 |
|  | [-0.035,0.069] | [-0.033,0.069] | [-0.036,0.069] | [-0.053,0.115] | [-0.085,0.081] | [-0.068,0.107] |
| Smoking | 0.514 | 0.539 | 0.519 | 0.197 | 0.234 | 0.310 |
|  | [-0.907,1.934] | [-0.872,1.951] | [-0.905,1.943] | [-0.813,1.206] | [-0.771,1.238] | [-0.680,1.301] |
| Alcohol | 0.039 | 0.036 | 0.033 | 0.126 | 0.141 | 0.127 |
|  | [-0.254,0.331] | [-0.259,0.331] | [-0.261,0.327] | [-0.339,0.592] | [-0.327,0.609] | [-0.341,0.594] |
| Age | -0.117^**^ | -0.112^**^ | -0.110^**^ | 0.136^*^ | 0.138^*^ | 0.137^*^ |
|  | [-0.221,-0.014] | [-0.217,-0.007] | [-0.214,-0.006] | [-0.003,0.274] | [-0.003,0.279] | [-0.004,0.277] |
| Type of Hukou | -0.186 | -0.193 | -0.193 | 0.664^***^ | 0.625^***^ | 0.574^**^ |
|  | [-0.467,0.095] | [-0.474,0.088] | [-0.474,0.088] | [0.221,1.107] | [0.175,1.075] | [0.121,1.027] |
| Ethnicity | -0.059 | -0.082 | -0.070 | 0.187 | 0.249 | 0.162 |
|  | [-0.377,0.260] | [-0.396,0.233] | [-0.391,0.250] | [-0.608,0.981] | [-0.528,1.026] | [-0.615,0.938] |
| Tree |  | 0.714 | 0.786 |  | -2.714^*^ | -2.108 |
|  |  | [-0.588,2.017] | [-0.548,2.120] |  | [-5.564,0.136] | [-4.900,0.685] |
| Bush | 0.137 |  | 0.347 | -6.865 |  | 3.292 |
|  | [-5.353,5.626] |  | [-5.069,5.764] | [-17.746,4.017] |  | [-6.716,13.299] |
| Grass | -0.281 | -0.285 |  | -1.562^*^ | -1.559^**^ |  |
|  | [-1.207,0.644] | [-1.186,0.616] |  | [-3.149,0.026] | [-3.036,-0.082] |  |
| Constant | 22.286^***^ | 22.288^***^ | 22.154^***^ | 17.829^***^ | 18.220^***^ | 17.693^***^ |
|  | [20.099,24.473] | [20.096,24.480] | [20.072,24.236] | [14.993,20.664] | [15.226,21.213] | [14.816,20.570] |
| N | 12221 | 12221 | 12221 | 9769 | 9769 | 9769 |
| F | 1.269 | 1.382 | 1.230 | 2.588 | 2.098 | 2.021 |
| R2 | 0.017 | 0.018 | 0.017 | 0.048 | 0.040 | 0.034 |

Note: *, **, and *** represent the p≤0.01, p≤0.05, p≤0.1, respectively. Units: Beta and [95% CI].
